# Longitudinal changes in the genetic and environmental influences on DNA methylation linked to obesity measures: a 5-year twin study

**DOI:** 10.1186/s43556-025-00334-y

**Published:** 2025-11-03

**Authors:** Xuanming Hong, Ke Miao, Weihua Cao, Jun Lv, Canqing Yu, Tao Huang, Dianjianyi Sun, Chunxiao Liao, Yuanjie Pang, Runhua Hu, Zengchang Pang, Min Yu, Hua Wang, Xianping Wu, Yu Liu, Wenjing Gao, Liming Li

**Affiliations:** 1 Department of Epidemiology and Biostatistics, School of Public Health, Peking University, Beijing, 100191 China; 2 Key Laboratory of Epidemiology of Major Diseases, Ministry of Education, Peking University, Beijing, 100191 China; 3 Qingdao Center for Disease Control and Prevention, Qingdao, 266033 China; 4 Zhejiang Center for Disease Control and Prevention, Hangzhou, 310051 China; 5 Jiangsu Center for Disease Control and Prevention, Nanjing, 210008 China; 6 Sichuan Center for Disease Control and Prevention, Chengdu, 610041 China; 7 Heilongjiang Center for Disease Control and Prevention, Harbin, 150090 China

**Keywords:** DNA methylation, Obesity, Genetic correlations, Longitudinal study, Twin study

## Abstract

The reproducibility of obesity-related DNA methylation (DNAm) sites (CpGs) remains low across studies, and the underlying mechanisms involving genetic and environmental contributions require further investigation. In this study, we systematically searched PubMed, EMBASE, and EWAS catalogue for CpGs associated with BMI, waist circumference (WC), and waist-to-hip ratio (WHR) from previous publications, identifying 29 studies with 2,603/892/28 CpGs for BMI/WC/WHR, respectively. Then, based on 1,074 twins from Chinese National Twin Registry, 493/149/8 CpGs were validated for BMI/WC/WHR, controlling for genetic factors. Following this, within the full twin population and a subsample of 308 twins with longitudinal data collected over a 5-year period, structural equation models (SEMs) were used to assess the heritability of CpGs and how it changes over time. The overall heritability of these obesity-related CpGs was relatively high, averaging 0.34 in full twin population, which decreased from 0.38 at baseline to 0.31 at follow-up. Bivariate SEMs were employed to investigate the genetic/environmental effects behind the longitudinal stability of DNAm. Genetic correlations between baseline and follow-up DNAm levels were high (mean = 0.74), whereas environmental correlations were relatively low (mean = 0.19), suggesting the role of genetic influence on the stability of DNAm at these CpGs. For longitudinal cross-relationships between obesity indicators and DNAm, considerably higher genetic contributions from baseline obesity traits to follow-up DNAm (mean = 0.15) was observed, compared to the baseline DNAm’s genetic influences on follow-up obesity indices (mean = 0.09, *P* < 0.01). These findings provide genetic evidence for obesity-related DNAm and demonstrate that the genetic effects of obesity have a persistent longitudinal influence on DNAm.

**Supplementary Information:**

The online version contains supplementary material available at 10.1186/s43556-025-00334-y.

## Introduction

Obesity is recognized as a major public health concern resulting yearly in significant disease and economic burden [1]. According to the diagnostic criteria for obesity in China, over half of the Chinese adult population was classified as overweight or obese in the 2019 nationwide survey [2]. There is an urgent need to elucidate the etiology of obesity for further prevention and control [3].

DNA methylation (DNAm), a dynamic and heritable epigenetic marker, primarily occurs in CpG dinucleotides (CpG sites) [4]. Many studies have explored the relationship between obesity-related traits and DNAm, and thousands of associated CpGs have been identified [5, 6]. Nevertheless, the reproducibility of CpG sites significantly associated with obesity across various studies remains insufficient, especially in Asian populations. The genetic variability among different study populations is likely a primary contributor to the observed discrepancies in these findings [7]. In this context, quantitatively assessing the genetic or environmental factors influencing both obesity and related methylation changes can provide novel insight into the genetic and epigenetic basis of obesity.

On the other hand, the causal relationship between obesity and methylation, as well as the underlying mechanisms, requires further exploration. BMI has been evidenced to precede changes in DNAm at multiple CpGs, which induces changes in expression of critical genes associated with the pathophysiology of several obesity-related diseases like diabetes and cardiometabolic diseases, such as *FTO* and *TNF* [4, 8–10]. Several studies based on Mendelian randomization have been conducted to explore the causal relationship between the obesity measures and DNAm, however, the conclusions reported based on different genes/CpGs are not consistent [11–14]. Methylation and obesity indicators are both dynamic measures that can change over time, highlighting the potential value of longitudinal study designs in elucidating the causal relationships between DNAm and obesity. One longitudinal research based on Bogalusa Heart Study and established cross-lagged models to explore the temporal relationship between DNAm and BMI. That research reported a unidirectional effect of BMI on DNA methylation at 25 CpG sites [4]. To date, no studies have investigated the genetic and environmental effects underlying this longitudinal influence. Quantitative assessment of the contributions of genetic and environmental factors to this longitudinal impact could enhance our understanding of obesity-related methylation changes, identify pathways of plasticity, and elucidating the temporal variability related biological mechanisms [15, 16].

Twin studies provide a valuable research tool in studying disease-related epigenetic changes, given that dizygotic twin pairs (DZ) share on average 50% of their segregating genes, while monozygotic twins (MZ) share 100% of their genes [17]. Through twin study, genetic or environmental factors underlying phenotypes can be controlled to some extent as confounding factors and can also be investigated to obtain the extent of those impacts. Based on twin population, a previous blood-based study has estimated the heritability of genome-wide DNA methylation, reporting a mean value of 0.19 and a median of 0.12 [18]. Several twin-based studies have applied bivariate structural equation models (SEMs) to evaluate the genetic and environmental effect shared between methylation at CpG sites and complex traits, including blood pressure and metabolic syndrome [19, 20]. Furthermore, twin studies facilitate the assessment of the contributions of genetic and environmental factors to the stability of methylation, as well as the longitudinal relationships between DNAm and obesity.

In this study, based on longitudinal data from the Chinese National Twin Registry (CNTR), our objectives were to: ①validate previously reported CpGs that associated with obesity-related indicators, which including BMI, waist circumference (WC), and waist-hip ratio (WHR) to obtain reproducible sites in Chinese population; ②investigate the heritability of these validated CpGs and the longitudinal changes in the heritability; ③explore the longitudinal contributions of genetic and environmental factors to the stability and changes of these validated CpGs; ④examine whether the genetic and environmental effects of obesity measures and methylation have a persistent mutual influence. Eventually, we can obtain insights into the genetic or environmental effects underlying obesity-related measures and their related epigenetic mechanism.

## Results

### Characteristics of the study population

Table 1 summarizes the detailed characteristics of the study participants [21]. Among 1,074 participants (mean age 49.9 ± 12.2 years), 758 were MZ twins. The average BMI was 24.8 kg/m^2^ (SD 3.9), while the mean WC and WHR were 86.9 cm (SD 10.8) and 0.9% (SD 0.1%), respectively. The within-pair correlations for BMI, WC, and WHR were found to be 0.6, 0.6, and 0.4 in MZ twins, versus 0.1, 0.2, and 0.2 in DZ twin subset, respectively.

**Table 1:** Characteristics of the analytic samples by study group

	Cross-sectional analysis	Longitudinal analysis		
	Total	Baseline	Follow-up	*P*-values
N	1,074	308		
Age, yrs	49.9 ± 12.2	50.2 ± 10.2	54.9 ± 10.2	< 0.01
Female, n (%)	341 (31.8)	121(39.3)		0.01
MZ, n (%)	758 (70.6)	186(60.4)		< 0.01
BMI, kg/m^2^	24.8 ± 3.9	24.2 ± 3.6	24.3 ± 3.5	0.02
WC (cm)	86.9 ± 10.8	75.4 ± 20.3	78.9 ± 18.3	< 0.01
WHR (%)	0.9 ± 0.1	1.0 ± 0.4	1.0 ± 0.3	< 0.01
Smoking status, n(%)				0.05
Current smoker	353(32.9)	95(30.8)	87(28.2)	
Former smoker5	140(13.0)	25(8.1)	35(11.4)	
Nonsmoker	581(54.1)	188(61.0)	186(60.4)	
Alcohol consumption, n (%)				< 0.01
Current drinker	456(42.5)	156(50.6)	85(27.6)	
Former drinker	73(6.8)	10(3.2)	27(8.8)	
Nondrinker	545(50.7)	142(46.1)	196(63.6)	

*MZ* monozygotic twins, *BMI* body mass index, *WC* waist circumference, *WHR* waist to hip ratio

### Validation of obesity-related measures associated CpG sites

To begin with, we performed a systematic search of the PubMed, EMBASE, and EWAS catalogue to identify EWAS studies on obesity-related measures and obtain the CpG sites that had been previously linked to obesity measures. The selection process, documented in Supplementary Fig. 1, adhered to Preferred Reporting Items for Systematic Reviews and Meta-Analyses (PRISMA) guidelines and outlines the literature screening process of identifying candidate CpG sites [22]. After the inclusion and exclusion criteria, a total of 29 articles were identified, including 3647/1269/44 CpG sites related to BMI/WC/WHR, respectively, which have been detailed in Supplementary Table 1. Among these, 2603/892/28 CpGs were present in our DNAm dataset, allowing for validation analysis.

The validation study was firstly conducted in the full study population, and 181 CpG sites were found to be significantly associated with BMI, 42 with WC, and 3 with WHR in this analysis (Supplementary Table 2). Among these sites, 11/1/0 sites revealed the opposite directions as in previous studies, respectively, and been removed from the next analysis steps. Subsequently, a separate validation analysis was also conducted within MZ twins (*n* = 756). Leveraging the MZ-paired twin design, the genetic and environmental backgrounds underlying the observed associations were further controlled. Through this analysis, DNAm difference within MZ twin pairs at 396 CpG sites were identified to be related with BMI, while for WC and WHR, the corresponding numbers of significant CpG were 113 and 6, respectively. Of these, 79/32/0 sites demonstrated discordant directions of association compared to previous reports. Since the analyzed independent and dependent variables represents a difference value between MZ twins rather than the original value, and the elimination of genetic and environmental confounding can lead to the revelation of the true direction of association, we highlighted these specific sites in the supplementary table, and retained them for the subsequent SEM analyses, nevertheless (Supplementary Table 3).

**Fig. 1:**
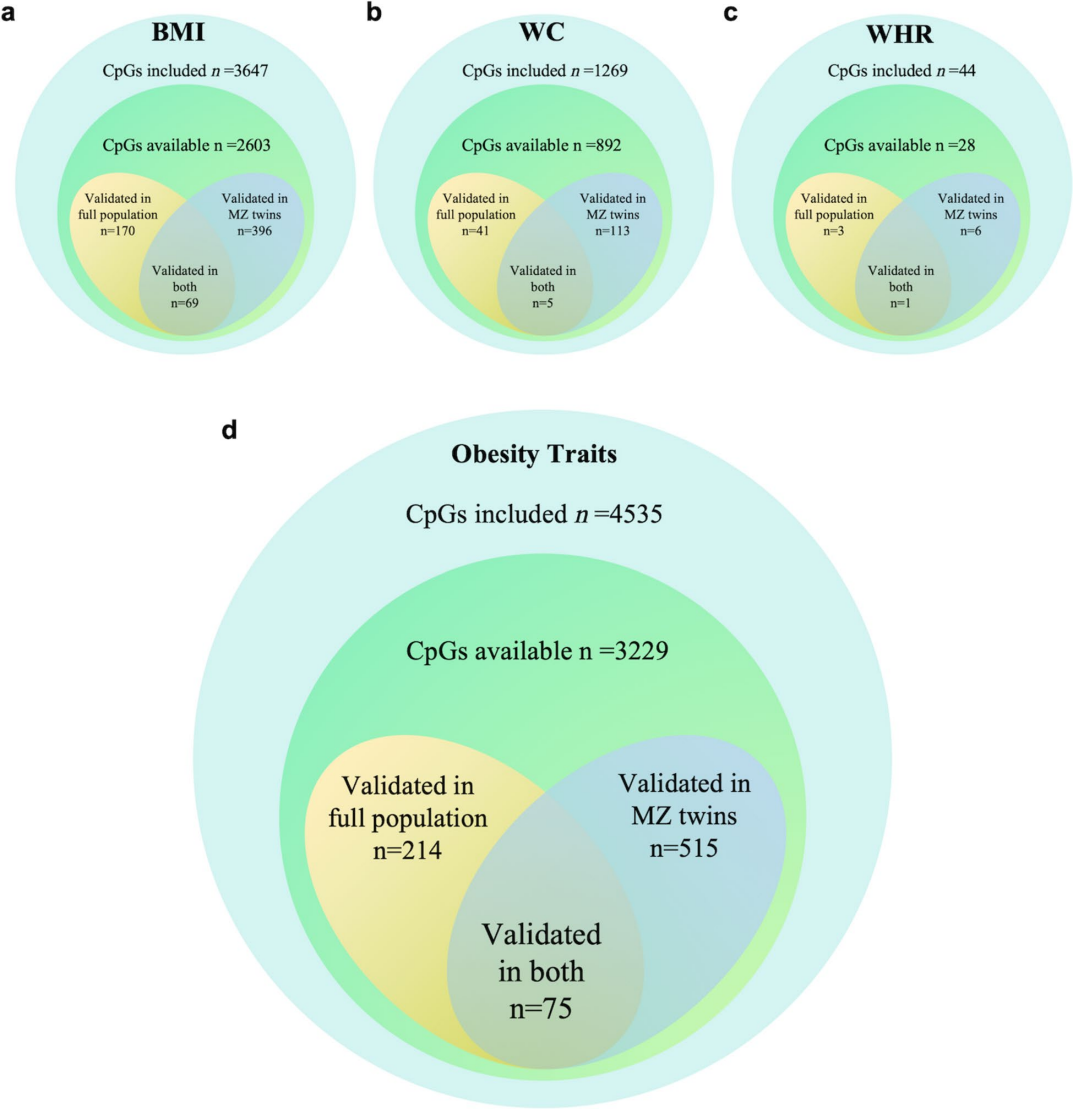
Characteristics and overlap of CpGs showing significance in the validation analysis. CpGs significantly associated with (**a**) BMI, (**b**) WC, (**c**) WHR, and (**d**) all obesity-related indices. CpG indicates 5′—cytosine—phosphate—guanine—3′ in DNA; BMI, body mass index; WC, waist circumference; WHR, waist to hip ratio

In total, 615 significant CpG sites were verified to be associated with obesity traits (after overlapping CpGs removal), including 493 sites for BMI (the sum from both the full-population analysis and the MZ-paired analysis), 149 sites for WC, and 8 for WHR (Fig. 1).

### Genetic and environmental variations in obesity traits related DNA methylation levels

In this study, the full analysis set comprised 1058 complete twins (including 378 MZ pairs and 151 DZ pairs), while the longitudinal subset included 151 twin pairs (93 MZ, 58 DZ pairs). Utilizing univariate structural equation models (SEMs), the heritability of all CpGs that were significant in the validation analysis was assessed, along with their longitudinal changes.

**Fig. 2:**
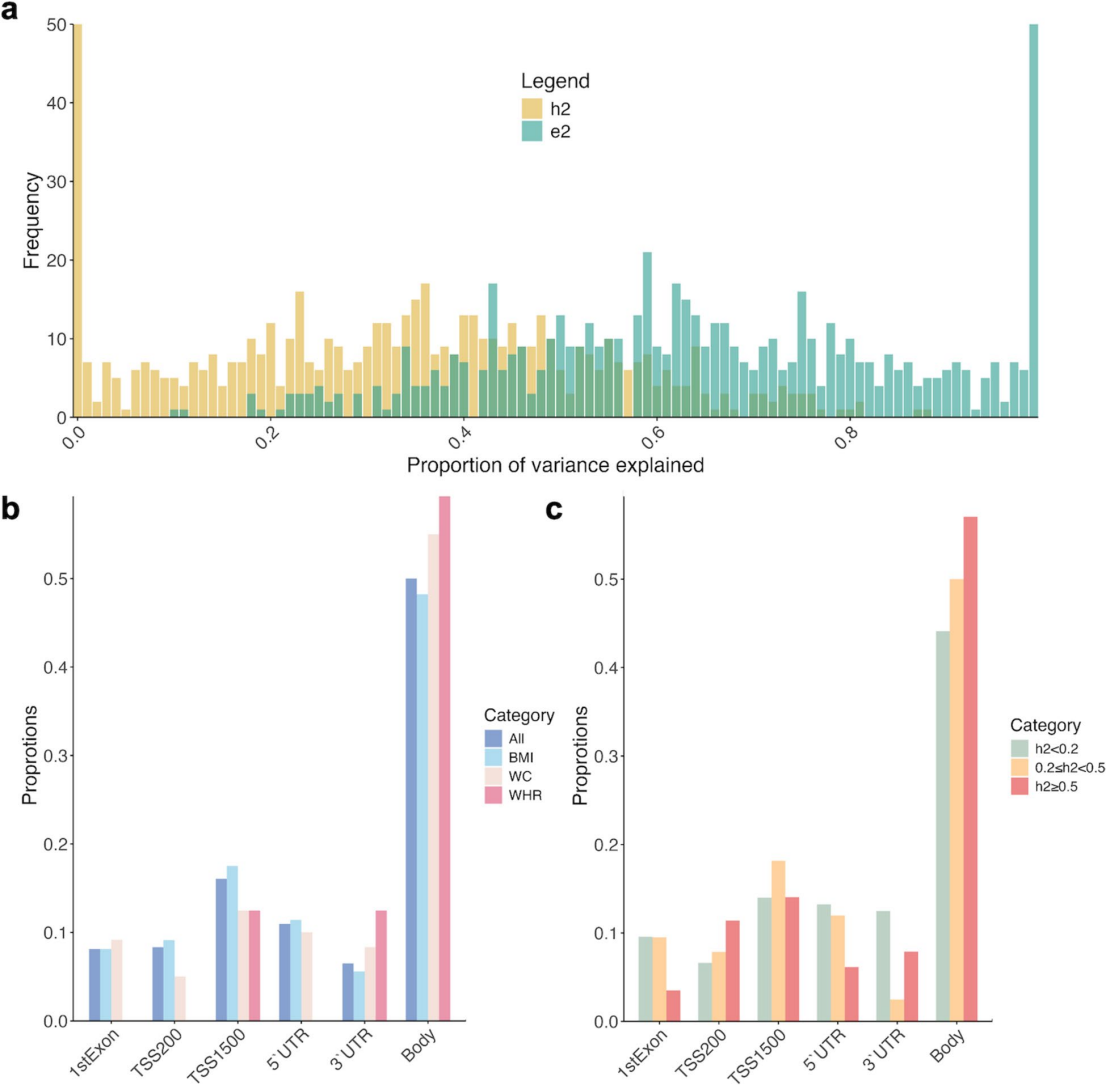
Genetic and environmental influences and distributions of obesity-related CpGs in full analysis population. (**a)** Histograms of univariate SEM model estimates for obesity-related CpGs at full analysis population (*n* = 1058); (**b**) Distribution of DNAm sites grouped based on obesity traits (*n* = 1058); (**c**) Distribution of DNAm sites grouped based on heritability (*n* = 1058). CpG indicates 5′—cytosine—phosphate—guanine—3′ in DNA; h^2^, heritability; e^2^, environmental components; BMI, body mass index; WC, waist circumference; WHR, waist to hip ratio

In the full study population, ACE models were fitted for 245 CpGs, and ADE models were fitted for 370 CpGs (Supplementary Table 4). In the full ACE/ADE models, the mean heritability estimated for all obesity-related CpG sites was documented at 0.34 (Table 2). The distributions of the heritability estimations were detailed in Fig. 2. The five CpG markers exhibiting the highest heritability estimates were cg26955383 (h2 = 0.89), cg03159676 (h2 = 0.88), cg01526748 (h2 = 0.82), cg09876440 (h2 = 0.82), and cg14476101 (h2 = 0.81), as shown in Supplementary Table 5.

**Table 2:** Twin correlations, heritability and variance components of DNAm at all obesity-related CpGs for baseline and follow-up

Parameter	Full analysis population				Baseline				Follow-up			
	Min	Median	Mean	Max	Min	Median	Mean	Max	Min	Median	Mean	Max
rMZ	−0.12	0.43	0.41	0.90	−0.16	0.51	0.49	0.93	−0.22	0.44	0.43	0.89
rDZ	−0.17	0.22	0.22	0.68	−0.17	0.28	0.28	0.81	−0.27	0.23	0.23	0.69
h2	0.00	0.35	0.34	0.89	0.00	0.41	0.38	0.93	0.00	0.32	0.31	0.89
Environmental component	0.10	0.65	0.66	1.00	0.07	0.59	0.62	1.00	0.10	0.68	0.69	1.00
Full Models												
	ACE(*n* = 245)				ACE(*n* = 304)				ACE(*n* = 304)			
a2	0.00	0.22	0.23	0.73	0.00	0.24	0.26	0.90	0.00	0.16	0.20	0.83
c2	0.00	0.11	0.12	0.47	0.00	0.17	0.20	0.74	0.00	0.17	0.19	0.70
e2	0.16	0.66	0.65	1.00	0.09	0.51	0.54	1.00	0.14	0.61	0.61	1.00
	ADE(*n* = 370)				ADE(*n* = 309)				ADE(*n* = 309)			
a2	0.00	0.09	0.16	0.71	0.00	0.07	0.15	0.81	0.00	0.01	0.13	0.67
d2	0.00	0.24	0.24	0.77	0.00	0.36	0.35	0.88	0.00	0.25	0.28	0.81
e2	0.10	0.58	0.59	1.00	0.07	0.47	0.49	1.00	0.10	0.58	0.59	1.00

*rMZ* correlation between MZ twins, *rDZ* correlation between DZ twins, *h*^*2*^ heritability, *a*^*2*^ additive genetic variance component, *c*^*2*^ common environmental variance component, *d*^*2*^ nonadditive genetic variance component, *e*^*2*^ unique environmental variance component

**Fig. 3:**
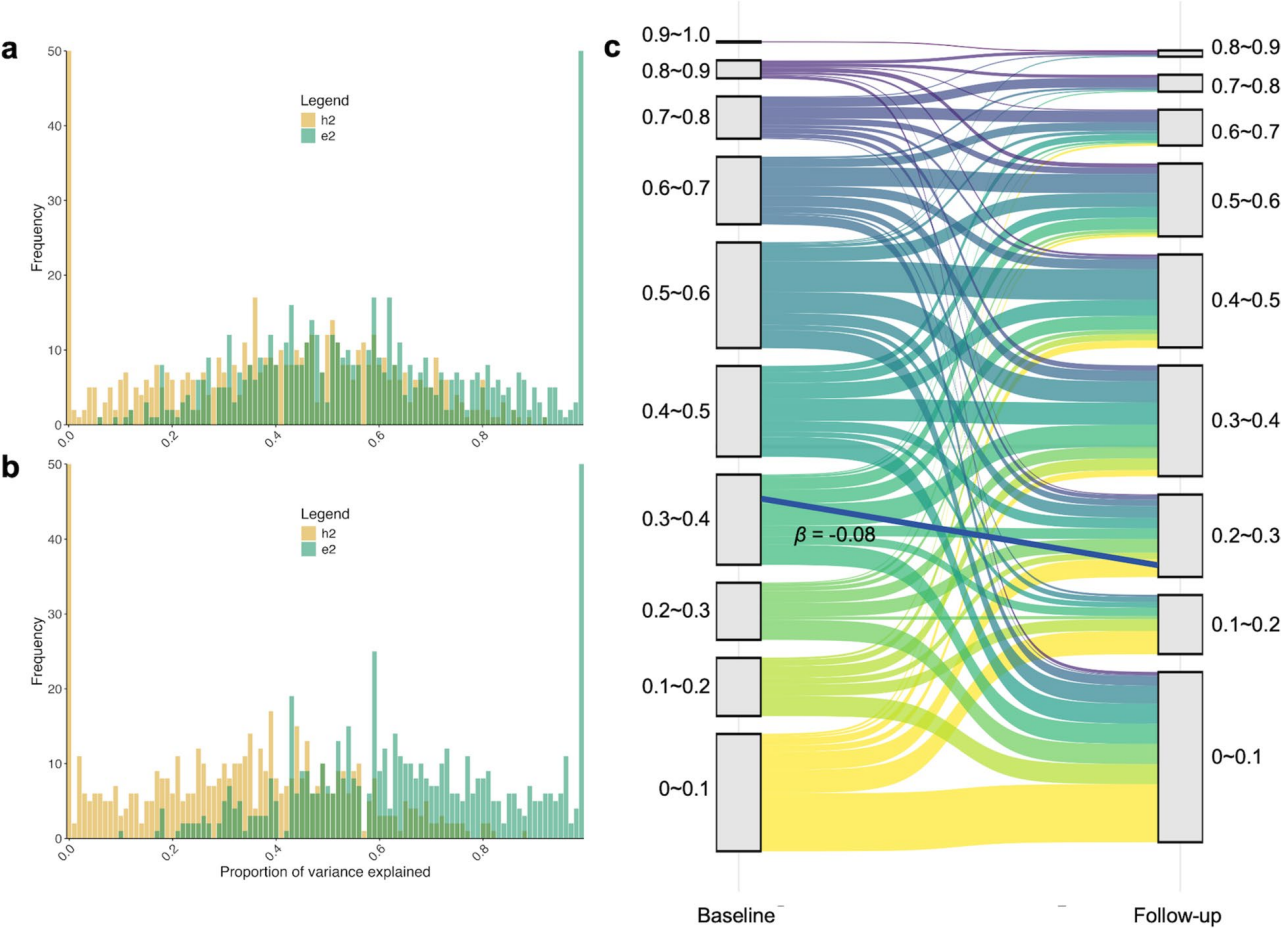
Genetic and environmental influences of obesity-related CpGs across 5 years. (**a)** Histograms of univariate SEM model estimates for obesity-related CpGs at baseline (*n* = 308); (**b**) Histograms of univariate SEM model estimates for obesity-related CpGs at follow-up (*n* = 308); (**c**) Sankey chart of longitudinal changes in heritability for obesity-related CpGs at follow-up (*n* = 308). CpG indicates 5′—cytosine—phosphate—guanine—3′ in DNA; h^2^, heritability; e^2^, environmental components

The longitudinal data includes 613 successfully validated sites. At baseline, 304 sites were fitted with ACE models, whereas 309 sites were modeled using ADE models. In the follow-up data, similarly, 304 sites fitted the ACE models, whereas 309 sites were modeled using an ADE model (Supplementary Table 6). In the full ACE or ADE models, the average heritability of the obesity traits related CpG sites were 0.38 at baseline and 0.31 at follow up (Fig. 3a and b). Compared to the heritability derived from the full population (cross-sectional heritability), the heritability calculated on baseline and follow-up data both exhibited significant overall differences (*P* < 0.01 for estimates from both periods). To note, cg03159676 was revealed to exhibit the highest heritability both at baseline (0.93) and the follow-up (0.89).

Figure 3c illustrates the detailed longitudinal variations in the heritability to the DNA methylation levels of 613 associated sites over five years. Specifically, a decrease in heritability was observed among 369 sites while an increase was observed among 206 sites. Then, the homogeneity tests were conducted, constraining the baseline genetic variance components to the follow-up to evaluate whether these changes were statistically significant. In these tests, 143 sites with a decrease in heritability and 51 sites with an increase were observed to be significant.

We also conducted t-tests to compare the CpG sites with and without significant changes in heritability. For sites exhibiting increased heritability, there was no significant difference in baseline heritability between those with significant and non-significant increases (*P* = 0.19). Conversely, for sites demonstrating a decrease in heritability, those with significant reductions exhibited higher baseline heritability compared to those with non-significant reductions (*P* < 0.01). This suggests that sites with higher baseline heritability are more prone to a downward trend in heritability.

**Fig. 4:**
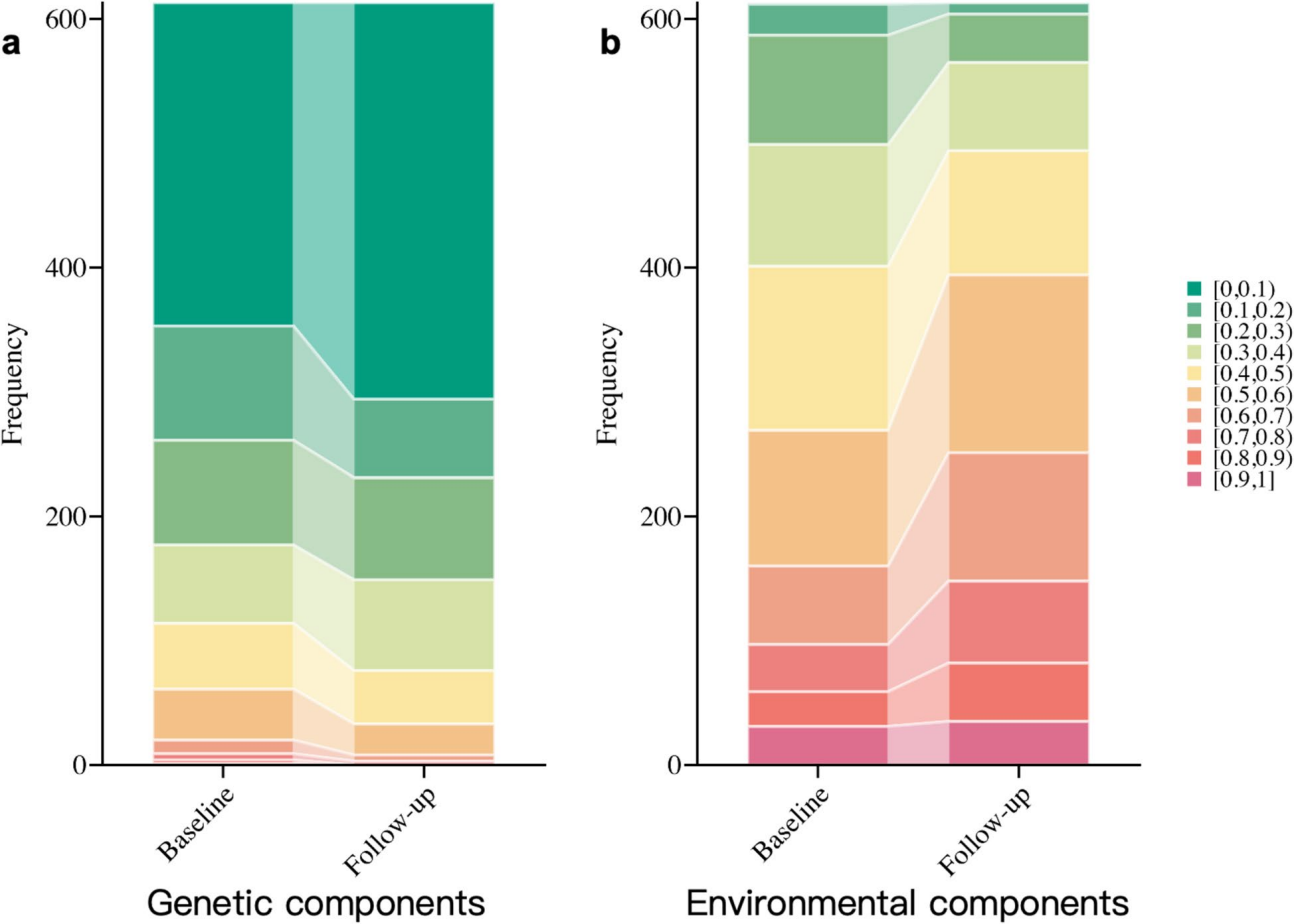
Changes in genetic and environmental variance of obesity-related CpGs across 5 years. (**a)** Sankey chart of longitudinal changes in genetic components for obesity-related CpGs (*n* = 308); (**b**) Sankey chart of longitudinal changes in environmental components for obesity-related CpGs (*n* = 308)

Through detailed observation on the variance components, we observed that the decline in heritability can be attributed to two key factors: a relative increase in environmental contributions (C + E, rising from 0.59 to 0.66, with a statistically significant t-test *P*-value < 0.01) and a concurrent decrease in genetic factors (A + D, declining from 0.37 to 0.29, also supported by a significant t-test *P*-value < 0.01), underscoring the growing influence of environmental effects on DNAm (Fig. 4, Supplementary Table 7).

### Longitudinal changes in genetic and environmental components underlying DNAm-obesity associations

To characterize the genetic or environmental contributions to the stability in the obesity-related DNAm between 2013 and 2018, bivariate SEMs were conducted to analyze the relationship between DNAm at baseline and at follow-up. The detailed information of genetic correlations (Ra) and environmental correlations (Re) between baseline and follow-up methylation levels, and path coefficients in SEMs are provided in the Supplementary Table 8. Collectively, the average Ra was 0.74 between baseline and follow-up DNAm, while the mean Re was 0.19, suggesting that a larger enduring impact of genetic effects on methylation, compared to a relatively smaller longitudinal effect of environmental factors.

**Fig. 5:**
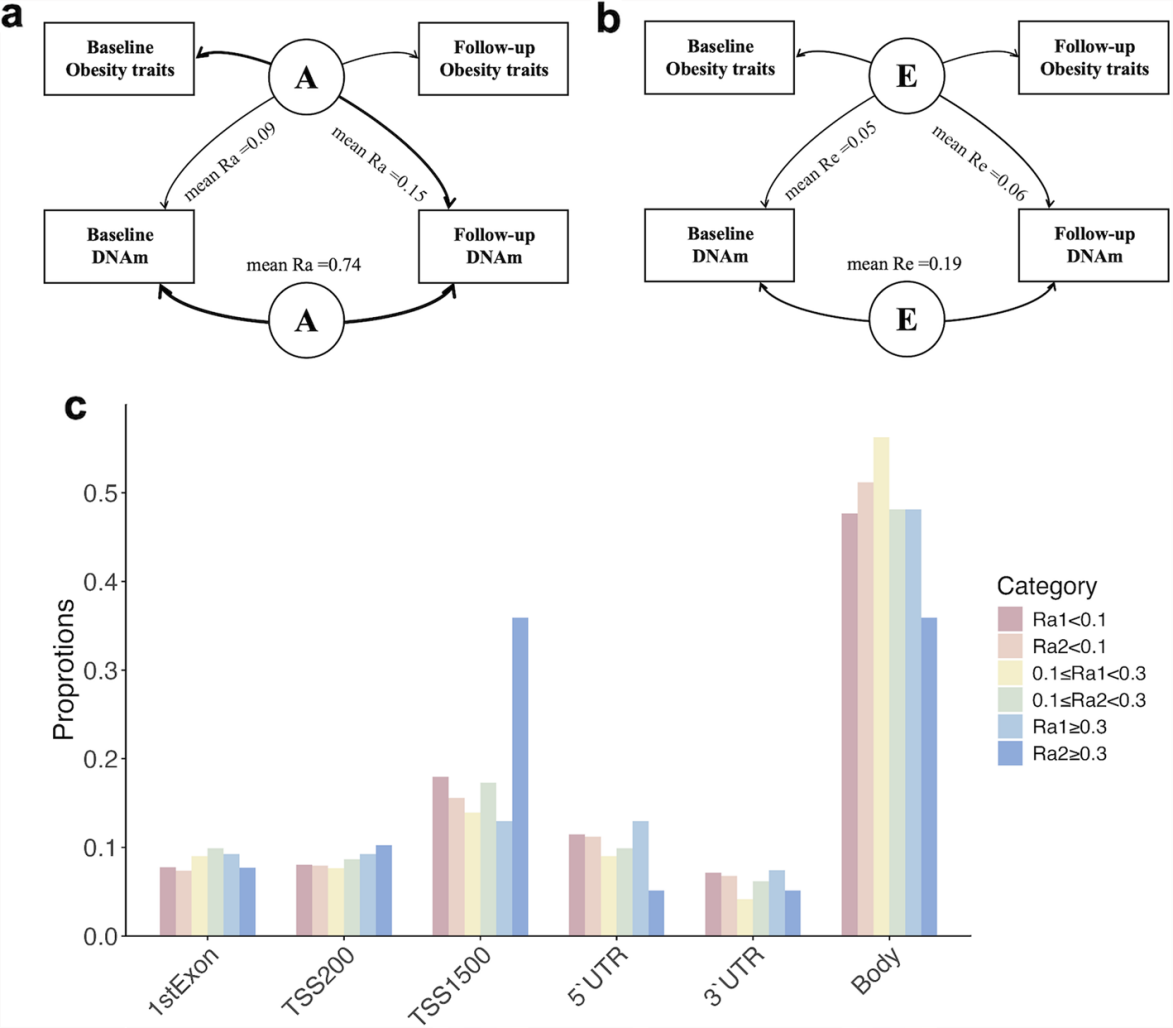
Longitudinal genetic and environmental correlations between obesity traits and DNAm and distributions for CpGs have different genetic correlations with obesity traits. (**a)** Longitudinal genetic correlations between obesity traits and DNAm for obesity-related CpGs (*n* = 308), "A" in the circle denotes the genetic contributions; (**b**) Longitudinal environmental correlations between obesity traits and DNAm for obesity-related CpGs (*n* = 308), "E" in the circle denotes the environmental contributions; (**c**) Distribution of DNAm sites grouped based on longitudinal genetic correlations with obesity traits (*n* = 308): Ra1: genetic correlations between baseline obesity traits and follow-up DNAm levels; Ra2: genetic correlations between baseline DNAm levels and follow-up obesity traits. DNAm, DNA methylation; Ra, genetic correlation; Re environmental correlation

Next, genetic and environmental correlations were estimated and compared between baseline obesity indices (BMI/WC/WHR) and follow-up methylation with those between baseline DNAm and follow-up obesity indices, using bivariate SEMs (Supplementary Table 9 and 10). The finding revealed that the genetic contribution of follow-up DNAm derived from baseline obesity traits (mean absolute Ra = 0.15) is considerably higher than that between baseline DNAm and follow-up BMI/WC/WHR (Ra = 0.09, *P* < 0.01 by t-test). For better comparison, the Ra between baseline obesity indices and DNA methylation, as well as between follow-up obesity indices and DNA methylation were additionally calculated. The resulting mean absolute Ra values were 0.07 and 0.08, respectively, and both were significantly lower than that between baseline obesity traits and subsequent DNA methylation (*P* < 0.01 by t-test for both, Supplementary Table 11 and 12). These findings suggests a more persistent genetic influence of obesity phenotypes on DNAm levels over time (Fig. 5).

On the other hand, the environmental components that from baseline BMI/WC/WHR have similarly been revealed to represent a relatively larger longitudinal impact on follow-up methylation (mean absolute Re = 0.06) than that from baseline DNAm on follow-up obesity traits (Re = 0.05, *P* = 0.04 by t-test).

## Discussion

Overall, we reported four main findings. First, we systematically searched the PubMed, EMBASE, and EWAS catalogue for previous reported BMI/WC/WHR-associated CpGs and conducted a validation study for these CpG sites. A total of 493 CpGs for BMI, 149 for WC, and 7 for WHR were eventually verified in this study. Second, based on SEMs, we estimated the heritability of these CpGs and their changes over time, which was 0.34 in the full analysis population, while decreased from a baseline average of 0.38 to 0.31 at follow-up. Third, we found a high genetic correlation between the baseline and follow-up methylation levels at these CpGs (mean Ra = 0.74). Furthermore, we observed a significantly greater genetic and environmental correlations between baseline obesity traits and DNAm at follow-up than those between baseline DNAm and follow-up obesity traits.

Although thousands of related-CpG sites been previously reported for obesity, these significant CpGs have a low degree of reproducibility. This might be caused by the difference in study settings, sample size, ethnicity and genetic diversity among studies [23–25]. To confirm the credibility of the associations and provide evidence from Chinese population, the present study validated the previously reported associations between DNAm and obesity indices in the Chinese twin population.

Among the validated sites, 211 were found to have been reported in more than one published article, further validating the robustness of our research findings (Supplementary Table 2 and 3). Several of these sites have been demonstrated to epigenetically participate in the pathogenesis of obesity and obesity-related metabolic disorders, including cg06500161, cg00574958, and cg11024682 [13, 26–28]. The *ABCG1* (annotated from cg06500161) encodes a member of the ABC transporter family, functionally, it is promotes cholesterol and phospholipid efflux from macrophages to high-density lipoprotein (HDL) particles [29]. Prior studies have demonstrated that methylation at cg06500161 can partly mediate the effect of BMI on HDL to some extent, implying that obesity-related metabolic consequences may operate through the modulation of methylation levels [30]. The methylation of cg00574958 has been demonstrated to regulate the expression of carnitine palmitoyl-transferase 1a (*CPT1A*) and plays a vital role in maintaining blood glucose levels and lipid metabolism [31–33].

**Fig. 6:**
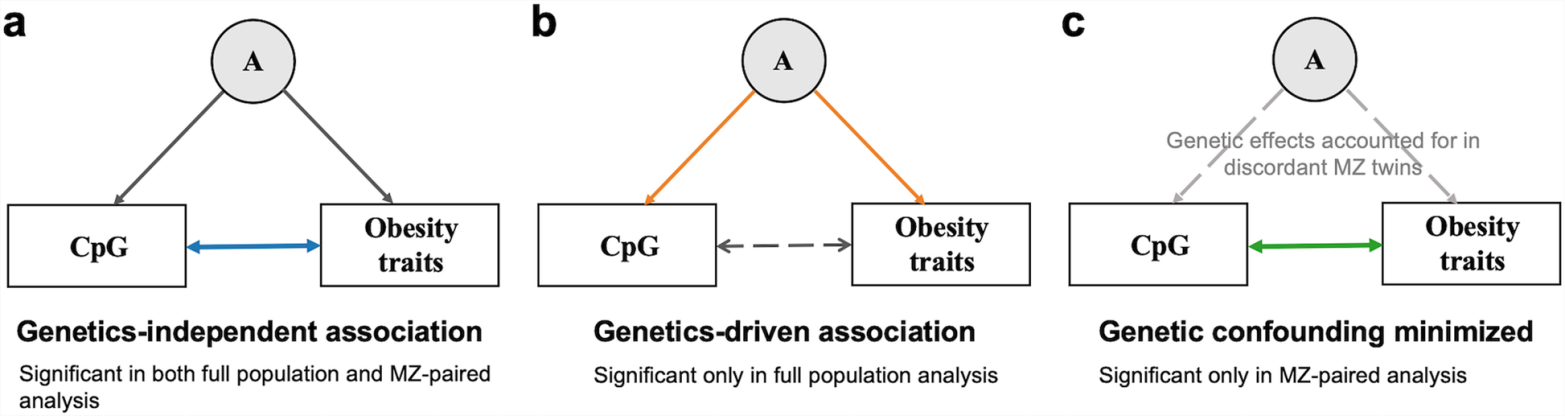
Potential explanations from association analyses. Figure a to c illustrate the potential results from association analyses. **a** The association between CpG and obesity traits is independent of genetic effects, as indicated by CpGs that show significance in both full population and MZ discordant analysis; (**b**) Genetic effects primarily drive the association between CpG and obesity traits, as evidence by CpGs that exhibit significance only in full population analysis; (**c**) Genetic effects confound the association between the CpG site and the obesity traits, as suggested by CpGs that show significance only in MZ discordant analysis. "A" denotes the genetic contributions

Notably, many significant CpGs that were absent from the analysis in full population were found in the discordant MZ twins analysis. One plausible explanation is that the paired design of MZ analysis increased statistical power for the results. The other interpretation is that, in the context of MZ analysis, where genetic information is controlled, the associations identified are more likely to be independent of genetic factors compared to those found in the full population. By comparing the results from these two analyses, we can obtain a deeper understanding of the role genetic factors play in the association between obesity and DNA methylation (Fig. 6). For instance, cg06500161 and cg00574958 both demonstrated significance in the MZ analysis. However, only cg06500161 was significant in the full analysis set. This suggests that while the association between cg06500161 and obesity traits may be influenced by genetic factors, their associations remain valid when considered independently of genetic influences [34].

On the other hand, the differences in methylation have been well documented to be caused by both heritable and environmental factors [35]. In our study, we investigated the heritability of validated obesity-related sites and how it changes longitudinally. Compared to the previously reported genome-wide average heritability of DNA methylation (0.19), a higher heritability was observed for these sites (mean = 0.34) [18]. We cross-referenced these CpGs with methylation quantitative trait loci (meQTLs) derived from a previous study. The reference study cataloged 11,165,559 high-confidence SNP–CpG associations (meQTLs) for 70,709 CpG sites, based on data from 3,799 Europeans and 3,195 Asians. Firstly, among the 615 validated CpGs, 231 were identified to have meQTLs, whereas in the total DNAm dataset of 374,968 sites, 62,701 sites displayed meQTLs (*P* < 0.01 by Fisher’s exact test). This may indicate that obesity-associated CpGs are more likely to be influenced by genetic factors. It was also demonstrated that CpG sites with validated meQTLs exhibited significantly higher mean heritability in this study, compared to those without meQTL (0.45 vs. 0.27, *P* < 0.01) [36]. This finding further substantiates the validity of the relatively high heritability estimates of DNAm that associated with obesity in this study.

In the following, based on longitudinal data, we found that over the past five years, there has been a significant decline in the average level of heritability of these CpGs. The study by Gaunt et al. similarly observed a decline in both the genetic influences on genome-wide methylation and the number of meQTLs with aging, emphasizing the increasing influence of environmental or stochastic factors [15]. Although there is a general decline trend in heritability, it was significantly increased at 51 sites, including the previously mentioned cg00574958 and cg11024682. This increase may be attributed to the fact that the expression of certain obesity-related genes can vary with increasing age [37]. The average age of the study population in this research is approximately 50 years. This age is also recognized as a pivotal time point at which significant changes in expression on many genes have been observed, as it coincides with the onset of various aging-related physiological alterations and the hormonal and endocrine changes associated with menopause in women [38–41]. Future studies and trials could prioritize older adults to investigate whether targeted modulation of methylation at these CpGs is feasible and whether such interventions could mitigate the obesity-related adverse outcomes.

In our study, we also observed a high genetic correlation between baseline and follow-up methylation at obesity-associated CpGs (0.74), compared to a lower environmental correlation of 0.19. The genetic correlation was higher than previous findings: a study that based on twins conducted bivariate SEMs between baseline and follow-up methylation epigenome-widely and reported a mean Ra of 0.27 and 0.20 for ADE and ACE models, respectively [16]. One possible explanation for this discordancy is the different time intervals between baseline and follow-up, which was 10 years in the previous study. Alternatively, this difference may be attributed to the higher genetic stability of validated obesity-associated CpGs. Our findings suggested that the stability of methylation is likely to be primarily driven by genetic factors. Environmental effects on methylation were non-stable in our study, i.e., environmental correlations between DNAm at baseline and follow-up were small, suggesting environmental influences were specific to each survey. A previous longitudinal twin study investigated the genetic and environmental correlations between baseline and follow-up measurements for both Horvath and Levine clocks. Similarly, it was found that all the person-specific environmental influences were unique to each measurement occasion [42]. This observation may reveal a common phenomenon in methylation research, wherein the impact of environmental factors exhibits substantial variability.

In another respect, the causal relationship between obesity-related indices and DNA methylation has been explored by adopting different statistical methods, yielding conclusions that support the influence of BMI on methylation at specific genes or CpGs [4, 11, 12]. However, supporting evidence from longitudinal design is still lack for further confirmation. In this study, although the causal relationship between them was not further validated, we provide novel evidence on driving factors behind these associations. Our results demonstrated significantly larger genetic correlations between baseline obesity indicators and follow-up DNAm compared to those between baseline DNAm and follow-up obesity indicators, revealing that the influence of obesity on DNAm may partly arise from the effects of obesity-associated genetic factors. These findings provide genetic evidence for the longitudinal influence from obesity on DNAm. Lastly, it was found in this study that, there are limited shared environmental factors between obesity phenotypes and DNAm. This demonstrates that even for obesity-related CpGs, the environmental effects influencing them may operate independently of the obesity phenotype. This observation highlights the presence of additional environmental influences that warrant further investigation [43].

This study also has several limitations. First, due to the presence of racial and regional disparities and research design variations, certain associations were not significant in this study [44]. Second, the sample size used in longitudinal analysis was modest. Since twins are a rare research resource, there are many challenges in collecting twin pairs, thus the recruitment is largely voluntary. Therefore, the CNTR is not fully representative of all Chinese twins. Third, due to the extended time span of the longitudinal study, different profiling platforms (450 K and EPIC arrays) were utilized to assess DNAm levels, which may introduce measurement bias for certain CpGs. Here, we referenced a study that analyzed cross-platform correlations between these two arrays and found that the mean cross-platform correlation for the 615 significant CpGs in our study was 0.52, with 81 CpGs exhibiting correlations < 0.2. However, after excluding these 81 CpGs, the remaining 534 CpGs had a mean correlation of 0.87 [45]. To enhance the comprehensiveness of our findings, we retained these CpGs for subsequent analyses. Finally, these analyses can only indicate genetic and environmental influences but do not delineate the underlying biological pathways, which require further investigation.

## Conclusions

In conclusion, we validated 615 CpGs to be associated with obesity-related traits, with a high mean heritability of 0.34. The results of the longitudinal study demonstrate an overall decrease in the heritability of methylation levels at obesity-related CpGs (from 0.38 to 0.31), which can be attributed to both reduced genetic and increased environmental influence. Next, we reported a relatively high genetic correlations (mean Ra = 0.74) between baseline and follow-up methylation for the validated CpGs, compared to the environmental correlations (mean Re = 0.17), indicating the role of genes on the longitudinal stability at these CpGs. Furthermore, genetic and environmental correlations were stronger for baseline obesity indices with follow-up DNAm than for baseline DNAm with follow-up obesity indices. These findings provide critical genetic insights into the longitudinal impact of obesity on DNA methylation, advancing our understanding of obesity etiology and its associated health implications.

## Materials and methods

### Study population

All data were obtained from the CNTR, whose study design, data-collection procedures, and participant characteristics have been described in detail elsewhere [46]. The data acquisition process was conducted in twice 2013 and 2018, which includes questionnaires, physical examinations, and blood withdrawal. The protocol was approved by the Biomedical Ethics Committee of Peking University, Beijing, China. Written informed consent was obtained from every participant in accordance with institutional requirements and the principles of the Declaration of Helsinki (IRB00001052-13022, IRB00001052-14021, IRB00001052-22032).

Inclusion criteria included: (1) both twins completed questionnaires and physical examinations, with comprehensive information provided for each; (2) blood withdrawal were available and completed from both members of twin pairs; (3) twins that have completed at least one detailed investigation in 2013 or 2018. The exclusion criteria encompassed pregnant females and their co-twins. During the subsequential DNAm quality controls and statistical analyses, exclusion of one twin may also lead to the removal of the co-twin. The initial cohort for analysis comprised of a total of 1,088 individuals (mean age 49.9 ± 12.2 years). Among these individuals, longitudinal data were available for 318 participants (accounting for 29.2% of the entire study population), who completed both the 2013 and 2018 surveys.

### Measurement of obesity-related indicators, twin zygosity and covariates

At both the 2013 and 2018 surveys, two replicate measurements of height, weight, WC, and hip circumference (HC) were obtained from each sample. If the first two measurements for height, WC, or HC differed by > 1 cm, a third measure is required. The average values of the two closest duplicates were utilized for subsequent analysis. Body weight was measured using an MC-780 body composition analyzer. WC was measured at the mid-point between the lower rib and iliac crest by tapes. HC was measured at the maximal circumference below the waist. Then, BMI was calculated as mean measured weight (kg) divided by squared height (m^2^), while WHR was computed as WC divided by HC.

Zygosity of twin pairs was determined utilizing a 59-SNPs panel present on the Illumina Infinium methylation arrays. Twin pairs sharing > 90% identical SNPs were identified as monozygotic twins (MZ) [47]. 816 twins (408 twin pairs) were classified as MZ in this study.

Primary covariates in this study included age, sex, smoking and drinking status. Smoking was systematically classified as current, former, and never smokers [48]. Similarly, drinking was categorized as current, former, and never drinkers [49].

### DNA methylation measurement

Peripheral blood was collected immediately after the physical examination. Then, genomic DNA underwent a modification process to bisulfite utilizing the EZ DNA methylation kit (Zymo Research, Orange County, CA, USA). Illumina Infinium Human Methylation Bead chip (Illumina, San Diego, CA, USA) were then used to quantify whole-genome DNA methylation profiles. Specifically, both the 450K and EPIC arrays were utilized. For the 2013 survey, the 450K array was applied to 326 samples for cross-sectional analyses and 123 samples for longitudinal analysis (baseline). The remaining samples were all assessed by the EPIC BeadChip, including 762 cross-sectional samples and 314 longitudinal samples in 2018 survey (follow-up), as well as 191 samples from the 2013 survey for the longitudinal analysis (baseline).

The detailed procedure for methylation data extraction, quality control, quantile normalization, and adjustment of cell-type proportions is provided in the supplementary materials. Briefly, the methylation level at each CpG site is reported as a β-value, reflecting the average proportion methylated on a 0–1 scale (0 means fully unmethylated; 1 means fully methylated).

After quality control was completed, a total of 378,654 CpG sites that were present on both the 450K and EPIC arrays and 1,074 participants with DNAm data were retained for subsequent analysis. Moreover, 374,786 CpGs and 308 subjects with longitudinal information were also included. To illustrate, for participants with longitudinal data, their data at follow-up was used for validation analysis and full-population heritability analysis.

### Statistical analysis

**Fig. 7:**
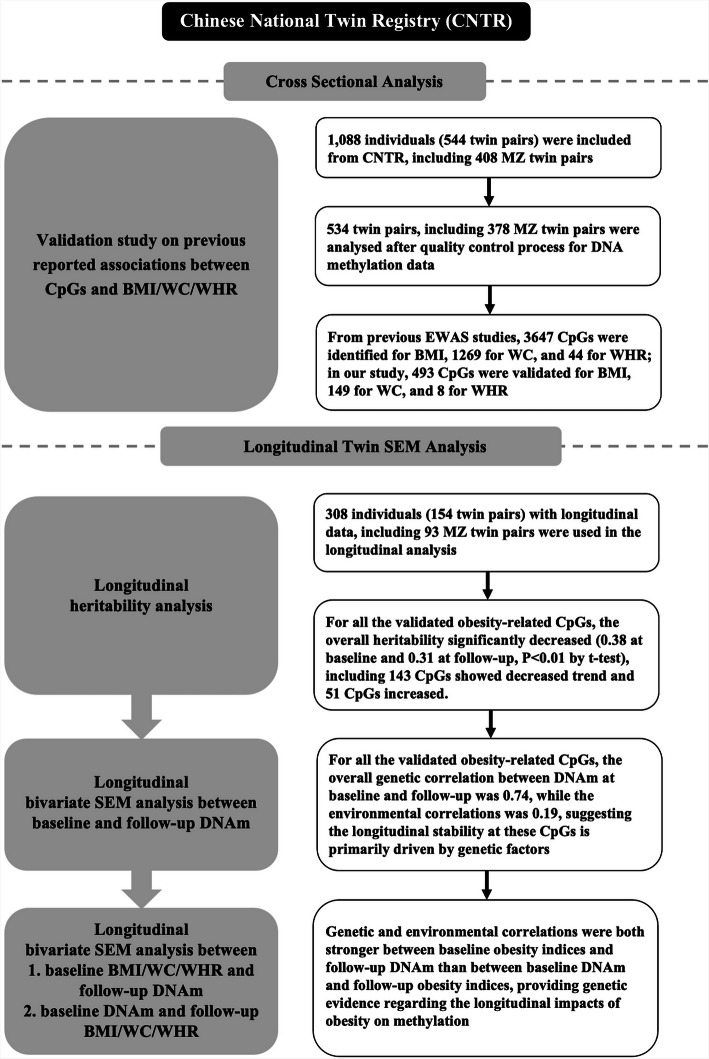
Study flow chart. CpG indicates 5′—cytosine—phosphate—guanine—3′ in DNA; BMI, body mass index; WC, waist circumference; WHR, waist to hip ratio; MZ, monozygotic twins; DNAm, DNA methylation; EWAS, epigenome-wide association study

The flow chart for analysis procedures was showed in Fig. 7. All analyses were conducted using R (R statistics), version 3.4.1.

### Validation of CpG sites for obesity-related indicators

In our study, we validated the CpG sites that had been reported to be associated with indicators for obesity. In this approach, we systematically selected relevant EWAS research for BMI/WC/WHR. Queried databases including PubMed, EMBASE, and the EWAS catalogue, restricting publications to those available up to July 20, 2025. The detailed search strategy and selection criteria for identifying relevant studies are provided in the supplementary materials.

A validation analysis for these candidate CpG sites was then conducted by using linear mixed-effect (LME) models in the full analysis population comprising 1,074 individuals. The DNAm β-values at each CpG site were treated as the continuous outcomes, while the obesity indicators were predictors. Covariates that consisted of age, sex, smoking and drinking status, as well as surrogate variables (derived from surrogate variable analysis), were introduced as fixed effects. The twin ID and zygosity information (MZ or DZ) shared by twin pairs were included as random intercept terms to account for the within-pair effects.

To further account for the genetic and environmental background underlying the associations, we conducted a discordant twin analysis specifically in MZ twins (within-pair analysis, *n* = 756). We also fitted LME models within MZ pairs to investigate the associations between the within-pair difference in BMI/WC/WHR and the within-pair difference in DNAm levels. The within-pair difference was computed by subtracting one twin’s trait value from that of their co-twin. Smoking status, and alcohol consumption of each individual were used as fixed effects terms, likewise, twin ID was included as a random effect to capture the correlations within twin-pairs. The formula of the model can be written as follows: Y_diff_ = X_diff_ + Covariate_self_ + TID(random effect term), the Y_diff_ and X_diff_ denote, respectively, the within-pair difference in the outcomes and predictors, defined as the individual’s value minus that of their co-twin [50]. To minimize the batch effects, we utilized the ComBat method in the DNAm data post-QC in the discordant MZ twin analyses.

Multiple testing was controlled using the Benjamini–Hochberg method (false discovery rate, FDR), with significance set at *P* < 0.05 in the validation study. CpG annotations were derived from the annotation file for Illumina Infinium EPIC array.

### Heritability estimations for the genetic and environmental determinants of DNAm associated with obesity-related measures

For all the verified obesity-associated CpG sites, we utilized the R package OpenMx (version 2.18.1) in the full twin population and twins with longitudinal data to estimate the relative contributions of genetic and environmental factors to the variance of DNAm [51].

We used structural equation models (SEMs) to estimate the relative contributions to DNAm variance in the full study samples and samples from 2013 and 2018, respectively. Only the twin pairs of same sex were included in the SEMs. The univariate structural equation models in the twin study have provided detailed description elsewhere [52]. Briefly, using the similarity between MZ and DZ twins, the models decomposed the phenotypic variation (i.e., variance) into four components: ①additive genetic variance, denoted as A (the cumulative effect of alleles); ②nonadditive genetic variance, denoted as D (an effect arising from the interaction between alleles); ③common environmental variance, denoted as C (variance resulting from shared environmental factors among twin pairs); ④and unique environmental variance, denoted as E (variance stemming from environmental factors that are not shared between twin pairs). Considering the difficulty in distinguishing between the C and D effects in twins raised together, either C or D, in addition to A and E, is generally included in the model [53]. We separately computed the ACE and ADE models, and selected either the ACE or ADE model to fit the data based on a comparison of their respective Akaike Information Criterion (AIC) values.

As illustrated in Supplementary Fig. 2, in the case of the ACE model, the difference between MZ twins is primarily attributable to unique environmental variance (E), since they shared 100% of their genetic background, thus the correlation observed between MZ twins were used as an assessment of (A + C). Conversely, DZ twins, who share an average of 50% of their genetic information, yield a correlation that directly estimates (½A + C). Based on these estimations, heritability serves as an approximation of the relative contributions of A (A + D for ADE models) [54]. We then fitted homogeneity models to test the equality of variance components in DNA methylation levels between 2013 and 2018. Poor model fit indicated statistically significant changes in these components over time.

In this study, age and sex were adjusted in all SEM models. The best model fit was selected based on one-sided P values and AIC values. One-sided tests with P < 0.05 were regarded as statistically significant.

### Longitudinal evaluation of the genetic and environmental determinants of DNAm and its association with obesity-related indicators

Longitudinal bivariate SEMs can be utilized to evaluate genetic and environmental contributions shared between two different variables, as well as contributions unique to each variable [42]. In this analysis, we first conducted bivariate SEMs to determine the degree to which shared genetic or environmental factors drove the stability and changes between baseline and follow-up DNAm of the CpG sites that associated with indicators for obesity. Comprehensive model details are provided elsewhere (see Supplementary Fig. 3). Similar to univariate SEM, phenotypic variation in the DNAm levels at the two temporal stages were decomposed into A, C, D, and E variations. For each site, we fitted ACE and ADE models separately and selected the optimal model by comparing their AIC values. Then, the genetic and environmental influence on the observed covariance between DNAm at two time points were identified by utilizing a sequence of submodels, which tested whether genetic, shared environmental, and unique environmental paths from baseline DNAm levels to that at follow-up can be constrained to 0. For example, if the shared genetic path from baseline to follow-up DNAm levels cannot be constrained to 0, it indicates the presence of an association or overlap between the genetic factors that influence the DNAm at both time points. The calculation of genetic and environmental correlations (Ra and Re) between the traits was further performed based on the variance and covariance matrix from the bivariate SEM, demonstrating the components shared between different temporal stages.

This longitudinal analysis also allows us to assess the temporal variations in the genetic and environmental influences on the associations between obesity-related indicators and DNAm. In this analysis, we employed separate analyses for the relationships between baseline BMI/WC/WHR and follow-up DNAm levels, as well as baseline DNAm and follow-up BMI/WC/WHR to investigate the persistent genetic effects of obesity phenotypes on DNAm and vice versa.

Prior to all SEM analyses, DNAm data underwent quantile normalization and were subsequently adjusted for cell-type proportions and batch effects using the ComBat approach.

## Supplementary Information

Supplementary Material 1.

Supplementary Material 2.

## Data Availability

In accordance with participants’ informed consent, which precludes disclosure of their data to third parties, the study datasets are not openly accessible. Data supporting the findings can be obtained from the corresponding author, Prof. Wenjing Gao, upon reasonable request.
