# Supplementary material for: Longitudinal changes in the genetic and environmental influences on DNA methylation linked to obesity measures: a 5-year twin study
Source: Mol Biomed. 2025 Nov 3;6:94. doi: 10.1186/s43556-025-00334-y (PMC12583361; doi:10.1186/s43556-025-00334-y)
Supplement: Supplementary file 1 — Supplementary Material 1. [file 43556_2025_334_MOESM1_ESM.docx]

**Longitudinal changes in the genetic and environmental influences on DNA methylation linked to obesity measures: A 5-year twin study**

**Author names and affiliations:**

Xuanming Hong, M.D.^a^, Ke Miao, M.D.^a^, Weihua Cao, M.D.^a^, Jun Lv, Ph.D.^a^, Canqing Yu, Ph.D.^a^, Tao Huang, Ph.D.^a^, Dianjianyi Sun, Ph.D.^a^, Chunxiao Liao, Ph.D.^a^, Yuanjie Pang, Ph.D.^a^, Runhua Hu, B.S.^a^, Zengchang Pang, B.S.^b^, Min Yu, M.D.^c^, Hua Wang, M.D.^d^, Xianping Wu, M.D.^e^, Yu Liu, M.D.^f^, Wenjing Gao, Ph.D.^a^, Liming Li, Ph.D.^a^

a Department of Epidemiology and Biostatistics, School of Public Health, Peking University, Beijing 100191, China; Key Laboratory of Epidemiology of Major Diseases, Ministry of Education, Peking University, Beijing 100191, China.

b Qingdao Center for Disease Control and Prevention, Qingdao 266033, China.

c Zhejiang Center for Disease Control and Prevention, Hangzhou 310051, China.

d Jiangsu Center for Disease Control and Prevention, Nanjing 210008, China.

e Sichuan Center for Disease Control and Prevention, Chengdu 610041, China.

f Heilongjiang Center for Disease Control and Prevention, Harbin 150090, China.

**Corresponding Author:**

**Wenjing Gao** - Email: [pkuepigwj@126.com](mailto:pkuepigwj@126.com); Postal address: Department of Epidemiology and Biostatistics, School of Public Health, Peking University, Beijing 100191, China; Phone numbers: +86 10 82801528 Ext 312

**Liming Li** - Email: [lmlee@vip.163.com](mailto:lmlee@vip.163.com); Postal address: Department of Epidemiology and Biostatistics, School of Public Health, Peking University, Beijing 100191, China; Phone numbers: +86 10 82801528 Ext 321

**Supplementary Text**

**DNA methylation data processing procedure**

Illumina Infinium Human Methylation 450K or EPIC Bead chip (Illumina, San Diego, CA, USA) were used in this study to quantify whole-genome DNA methylation profiles. Specifically, the 450K array was employed on 326 and 123 samples from the 2013 survey for cross-sectional and longitudinal analyses, respectively. The EPIC BeadChip was used for cross-sectional and longitudinal analyses on 762 and 314 samples, respectively, from the 2018 survey, as well as for longitudinal analysis of 191 samples from the 2013 survey. After blood collection, the samples were centrifuged and stored at -80°C until further processing. Methylation profiling was conducted in two phases: the first batch of DNA extracted in 2016 was analyzed using the Illumina 450K BeadChip, while the second batch extracted in 2020 was analyzed using the Illumina EPIC BeadChip. Probes from both the 450K and EPIC Bead Chips were retained for the analysis. The inter-assay reproducibility between the two BeadChips for the samples has been investigated to be 98%. Moreover, it was found that 90% of the probes present in the 450K microarray could be replicated using the EPIC BeadChip^1^. Samples from the Illumina EPIC and 450K platforms were merged using the “combineArrays” function in the R package “minfi” (version 1.34.0) to create a combinded dataset^2^.

Methylation levels of each CpG sites were reported as β-values, representing average methylation level ranging from 0 (fully unmethylated) to 1 (fully methylated). β-values were computed using the formula: β= M/(M+U+100), wherein M and U represent respectively for the average probe signal intensity at each site in the methylated and unmethylated states, which were assessed utilizing the R package “minfi”^2^.

Quality control (QC) measures were implemented for the DNAm data to filter out low-quality detection probes and samples from the DNAm dataset. In this study the measures included: (1) identification of missing probes that no significant differences were observed (P> 0.01) between the signals from the CpG site and the blank control from probes; (2) exclusion of probes with minor allele frequency (MAF) > 0.01 or those that had annotated single nucleotide polymorphisms (SNPs) on the microarray; (3) removal of missing probes with a detection P> 0.01 in more than 1% of the samples; (4) elimination of cross-reactive probes; (5) deletion of samples with missing rates exceeding 1% in probes.

Subsequently, the β-values that representing DNAm levels were quantile normalized, followed by adjustment for blood cell proportions (CD4T, CD8T, Mono, NK, Bcell, and Gran) through implementation of the “ChAMP” package (version 2.18.3)^3^. Furthermore, to minimize potential confounding effects introduced during the DNA methylation detections, data within each assay was corrected to account for experimental batch effects using the surrogate variable analysis function available in the R package “sva” (version 3.38.0)^4^. However, the ComBat approach in the R package “sva” was applied for all structural equation model analysis and discordant MZ analysis.

**Literature search strategy**

A systematic review was performed in the PubMed database using the following search terms.: (("Body Mass Index"[MeSH] OR "waist circumference"[MeSH] OR "Waist-Hip Ratio"[MeSH] OR BMI[tiab] OR Body Mass Index[tiab] OR waist circumference[tiab] OR waist circumference to hip ratio[tiab] OR Waist-Hip Ratio ratio[tiab]) AND (Methylome[tiab] OR epigenomewide[tiab] OR epigenome-wide[tiab]) OR EWAS[tiab]). The Embase database was searched by the following terms: ('BMI'/exp OR 'Body Mass Index'/exp OR 'waist circumference'/exp OR 'waist circumference to hip ratio'/exp OR 'WHR'/exp OR 'waist to hip ratio'/exp) AND ('Methylome'/exp OR 'epigenemewide'/exp OR 'ewas'/exp OR 'epigenome wide association study'/exp OR 'epigenome wide'/exp) AND ('article'/it OR 'article in press'/it OR 'preprint'/it OR 'short survey'/it). The EWAS catalogue was searched with the search terms: “BMI”, “body mass index”, “waist”, “waist circumference”, “waist circumference to hip ratio”, and “waist to hip ratio”.

The systematic searches were all performed until July 20th, 2025. We evaluated the efficiency of each publication's title and abstract in selecting candidate CpG sites. We identified 340 relevant publications in PubMed (1194), Embase (308) and EWAS catalogue (18). The epigenome-wide association study on BMI/WC/WHR were selected and included for the identified associations. Studies focused on irrelevant phenotypes, non-methylation-related investigations, research utilizing umbilical cord blood, placental blood, and saliva for DNA methylation sequencing, studies involving participants with severe diseases such as breast cancer, investigations related to obesity surgery/treatment, research examining the association between maternal obesity during pregnancy and offspring methylation levels, studies specifically targeting the methylation mechanisms of particular genes, articles lacking an introduction to EWAS methods, studies failing to define a cut-off threshold for significance levels, and ultimately studies that did not identify or report any significantly associated loci related to BMI/WC/WHR were excluded. In addition, systematic reviews, commentaries, abstracts, and editorial letters were also excluded from our analysis.

Each report was screened independently by two investigators (X.H. and K.M.), and disagreements were resolved by a third senior curator (W.G.). After completing the literature reading and screening, X.H. extracted data from the reports, including information on the study population, study design, DNA methylation detection methods, number of significant associations, phenotypes, direction of associations, effect sizes, p-values, covariates involved in the analysis, and methods for multiple testing correction (as listed in Supplementary Table 1).

**Supplementary Figures**


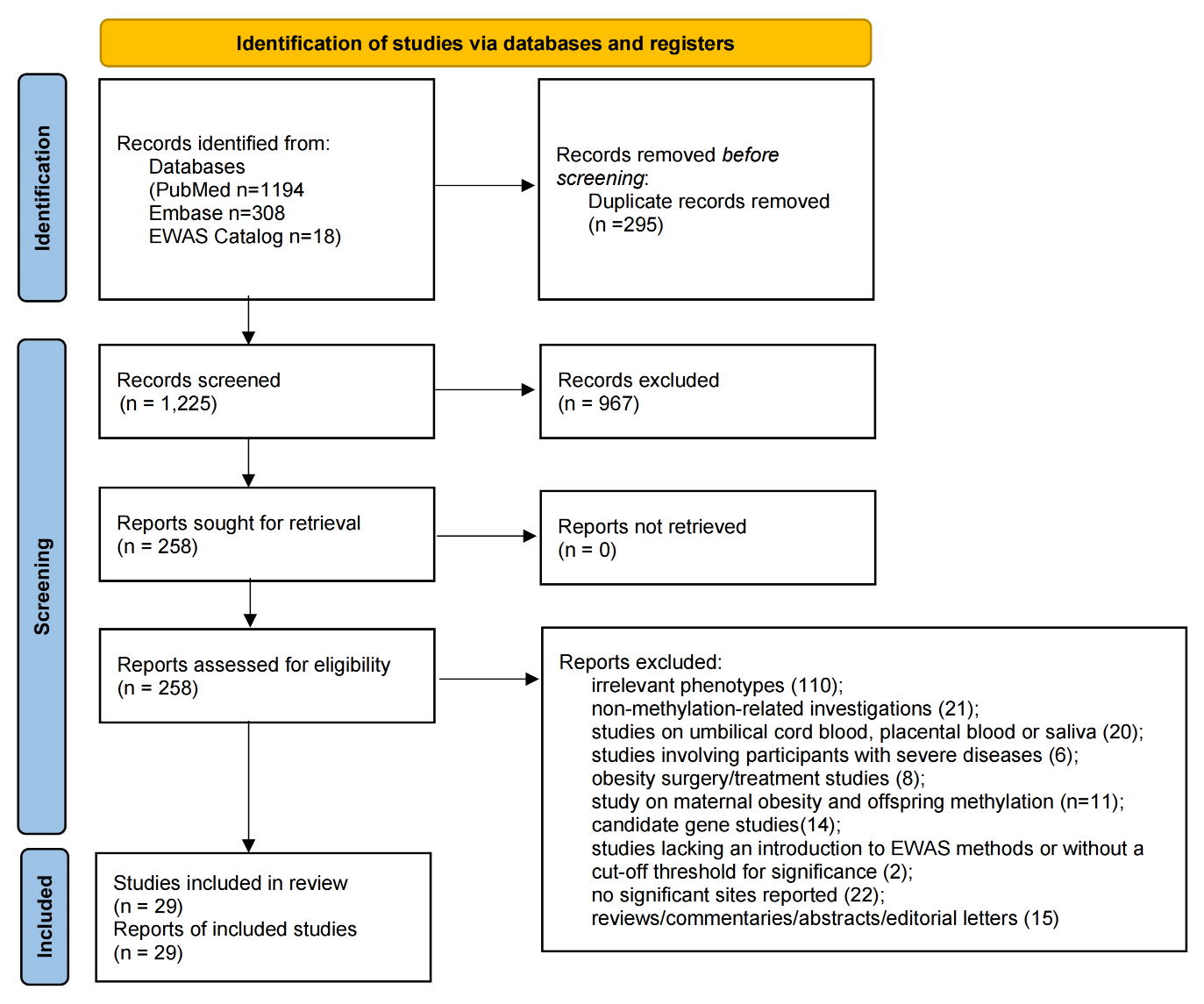


**Supplementary Figure 1. PRISMA 2020 flow chart of the study selection process**


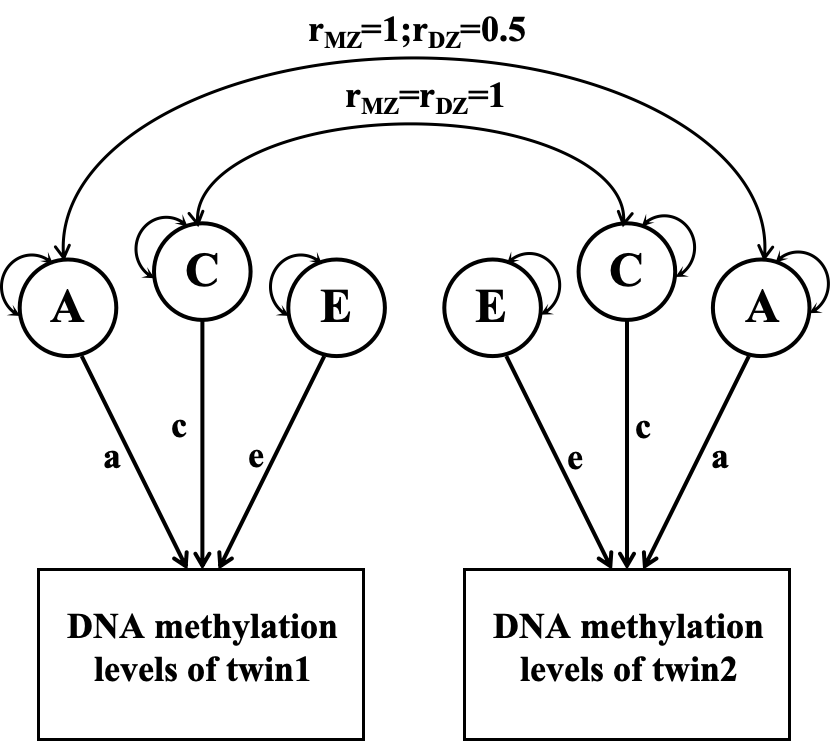


**Supplementary Figure 2. Diagram for the univariate structural equation model**

The phenotypes for a twin pair are depicted in squares, while latent factors are in circles. The correlations of additive genetic variance (A) are 1 in MZ twins and 0.5 in DZ twins. For both MZ and DZ twins, the correlations of common environmental variance (C) are 1. Unique environmental variance (E) is consistently 0.


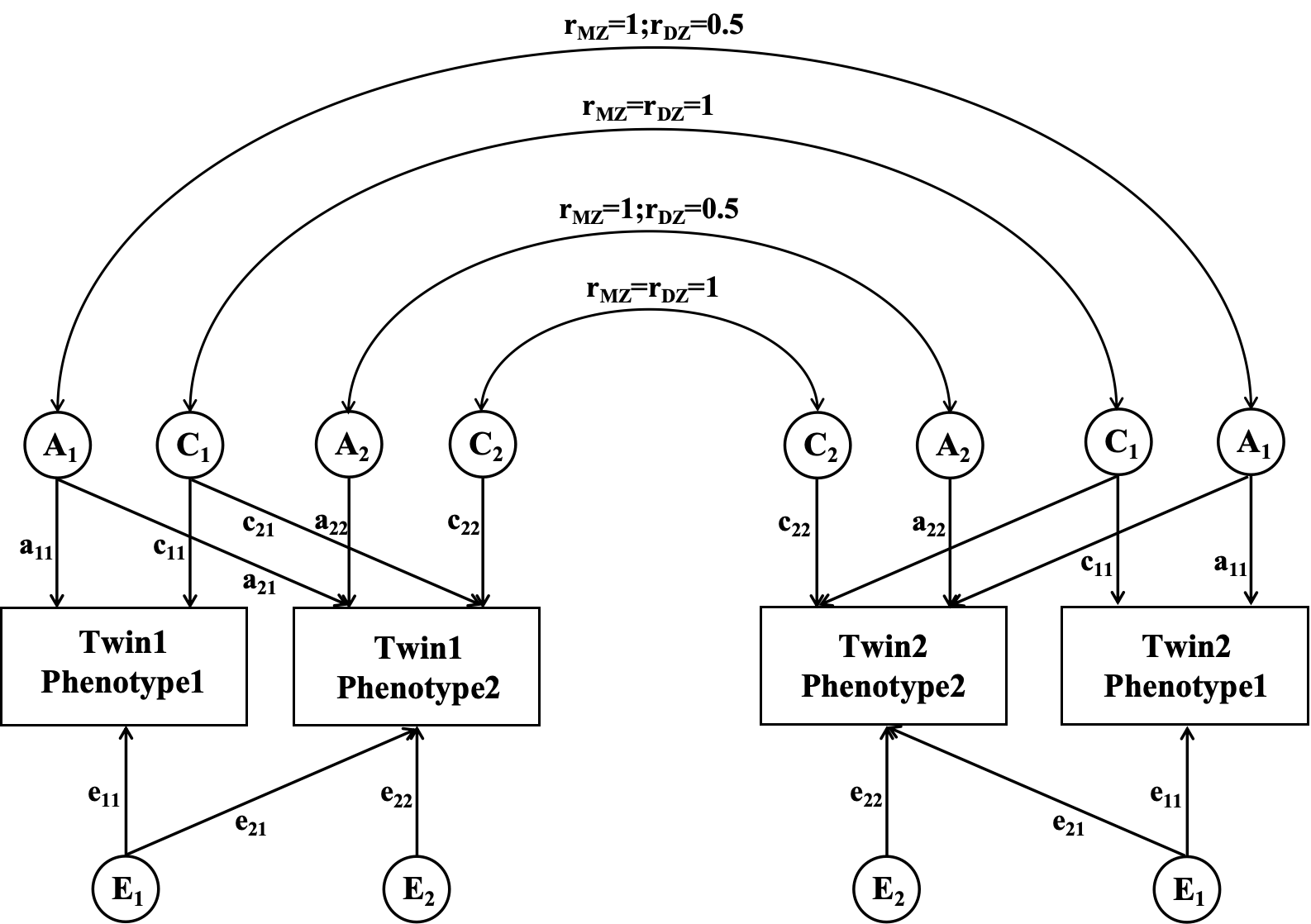


**Supplementary Figure 3. Diagram for the bivariate structural equation model**

The phenotypes for a twin pair are depicted in squares, while latent factors are in circles. The correlations of additive genetic variance (A) are 1 in MZ twins and 0.5 in DZ twins. For both MZ and DZ twins, the correlations of common environmental variance (C) are 1. Unique environmental variance (E) is consistently 0.

**Reference**

1. Pidsley R, Zotenko E, Peters TJ, Lawrence MG, Risbridger GP, Molloy P, et al. Critical evaluation of the Illumina MethylationEPIC BeadChip microarray for whole-genome DNA methylation profiling. Genome Biol. 2016;17(1):208.

2. Aryee MJ, Jaffe AE, Corrada-Bravo H, Ladd-Acosta C, Feinberg AP, Hansen KD, et al. Minfi: a flexible and comprehensive Bioconductor package for the analysis of Infinium DNA methylation microarrays. Bioinformatics. 2014;30(10):1363-9.

3. Tian Y, Morris TJ, Webster AP, Yang Z, Beck S, Feber A, et al. ChAMP: updated methylation analysis pipeline for Illumina BeadChips. Bioinformatics. 2017;33(24):3982-84.

4. Leek JT, Johnson WE, Parker HS, Jaffe AE, Storey JD. The sva package for removing batch effects and other unwanted variation in high-throughput experiments. Bioinformatics. 2012;28(6):882-3.
